# c.1263+1G>A Is a Latent Hotspot for CYP27A1 Mutations in Chinese Patients With Cerebrotendinous Xanthomatosis

**DOI:** 10.3389/fgene.2020.00682

**Published:** 2020-07-01

**Authors:** Jingwen Jiang, Guang Chen, Jingying Wu, Xinghua Luan, Haiyan Zhou, Xiaoli Liu, Zeyu Zhu, Xiaoxuan Song, Shige Wang, Xiaohang Qian, Juanjuan Du, Xiaojun Huang, Mei Zhang, Wei Xu, Li Cao

**Affiliations:** ^1^Department of Neurology, Ruijin Hospital and Ruijin Hospital North, Shanghai Jiao Tong University School of Medicine, Shanghai, China; ^2^Department of Neurology, The First People’s Hospital of Huainan, The First Affiliated Hospital of Anhui University of Science and Technology, Huainan, China; ^3^Department of Neurology, Shanghai Fengxian District Central Hospital, Shanghai Jiao Tong University Affiliated Sixth People’s Hospital South Campus, Shanghai, China

**Keywords:** Cerebrotendinous xanthomatosis, CYP27A1, clinical features, compound mutations, Chinese, hotspot

## Abstract

**Background:**

Cerebrotendinous xanthomatosis (CTX) is an autosomal recessive disorder of bile acid synthesis caused by mutations in the CYP27A1 gene. CTX is an underdiagnosed and potentially treatable disease, thus a detailed appreciation of the phenotypic spectrum and genetic characteristics are crucial for early diagnosis and treatment.

**Objectives and Methods:**

Four CTX families with mutations in the CYP27A1 gene were enrolled in our study. We investigated the clinical characteristics and molecular genetic features of the probands with CTX. Genetic analysis was performed for detecting gene variants. Sanger sequencing and segregation analysis were conducted for haplotype analysis.

**Results:**

All the four probands were compound heterozygote for two CYP27A1 variants, including one mutation in c.1263+1G>A (intron 7) splice site, two novel likely pathogenic mutations (c.255+1G>T and c.1561dupA) and three pathogenic mutations including c.379C>T, c.1263+1G>A and c.1537C>T previously reported. All of the subjects presented with spastic paraparesis. The other common clinical features included ataxia, childhood-onset diarrhea, cataracts, intellectual disability, tendinous xanthomas and dentate nuclei signal alterations at MRI.

**Conclusion:**

Two novel likely pathogenic mutations (c.255+1G>T and c.1561dupA) were reported in our study. The 1263+1G>A mutation was commonly seen in Chinese reported case series (7/25, 28%) and could be a latent hotspot for Chinese CTX mutations. Our study expanded the mutation spectrum of CYP27A1 gene and provide an insightful view of the phenotypic spectrum and genetic characteristics to help early diagnosis and treatment with to improve neurologic dysfunction.

## Introduction

Cerebrotendinous xanthomatosis (CTX) is a rare autosomal recessive bile acid disorder caused by the deficiency of the mitochondrial enzyme 27-sterol hydroxylase (CYP27A1). This enzyme is located on the inner membranes of the mitochondria, and plays an important role in bile acid synthesis pathways (Russell, 2003). The deficiency of CYP27A1 leads to attenuated cholic acid formation and decreased production of chenodeoxycholic acid (CDCA). Consequently, elevated level of cholesterol 7α-hydroxylase in the classic bile acid pathway resulted in increased quantities of 7α-hydroxy-4-cholesten-3-one, which was an efficient precursor of cholestanol (Nie et al., 2014). The increased cholesterol synthesis and enhanced production of cholestanol induced up-regulated plasma cholestanol. Abnormal deposition of cholestanol and cholesterol could be detected in tissues throughout the body, especially in crystalline lenses, cerebra and muscle tendons (Bjorkhem and Leitersdorf, 2005). CTX maybe underdiagnosed with a possibly prevalence of 1:40,000 to 1:400,000 because of non-specifical symptoms (Appadurai et al., 2015). The clinical picture is extremely heterogeneous. The patients with CTX usually present with neonatal cholestatic jaundice, chronic unexplained chronic diarrhea, bilateral cataracts, tendon xanthomas, and neurologic dysfunction (Mignarri et al., 2014). Brain magnetic resonance (MR) imaging of CTX patients commonly demonstrates cortical and cerebellar atrophy, white matter signal alterations like bilateral hyperintensities of the dentate nuclei (Mignarri et al., 2017). The biochemical features that distinguish CTX from other conditions included increased plasma level of cholestanol and normal-to-low plasma cholesterol concentration (Debarber et al., 2014). The diagnosis of CTX can be further confirmed by detecting mutations in the CYP27A1 gene.

Here, we described four cases of patients with delayed diagnosis of CTX showing various clinical features. Gene analysis of all these patients revealed a status of compound heterozygous mutations in the CYP27A1 gene.

## Methods

### Participants

Four probands (T4550, T4979, T5200, and T5300) identified with CTX were enrolled in this study. The patients were evaluated by clinical features and mutation analysis. The study had received ethics approval from the Ethics Committee of Ruijin Hospital, Shanghai Jiao Tong University School of Medicine, Shanghai, China. All the participants had signed informed consents, and parental permission of the subjects with cognitive dysfunction were obtained.

### Genetic Analysis

Blood samples of the patients and their parents are collected after obtaining informed consent. Genomic DNA was extracted by phenol-chloroform method. Whole exome sequencing was performed for 4 probands using Agilent SureSelect v5 reagents (Agilent Technologies, Santa Clara, CA, United States) and Illumina HiSeq X Ten platform (Illumina, San Diego, CA, United States). The human genome reference sequence (GRCh37/hg19) were used to map the sequence reads. On the basis of public databases of normal human variation (1000g, ExAC, and gnomAD), all the variants of which Minor Allele Frequency (MAF) is higher than 1% were filtered. Polyphen-2, SIFT, and Mutationtaster were used to predict the pathogenicity of mutation. American College of Medical Genetics and Genomics (ACMG) Guidelines were used to interpret and classify variants. Sanger sequencing was carried out to confirm the putatively pathogenic variants.

### SNPs for Haplotype Analysis

The SNPs used in the study for haplotypes were identified based on the dbSNP database^1^, human assembly GRCh37. SNPs were selected as follows (listed in the leftmost two columns of Table 1: (1) CHB (Han Chinese in Beijing, China) heterozygosity in the 1000 Genomes database; (2) MAF (minimum allele frequency) of CHB of 0.15-0.7, and (3) Upstream, downstream SNPs of c.1263+1G>A in CYP27A1. Sanger sequencing was performed to identify the SNPs.

**TABLE 1:** SNP-based genotype and haplotypes of the four families.

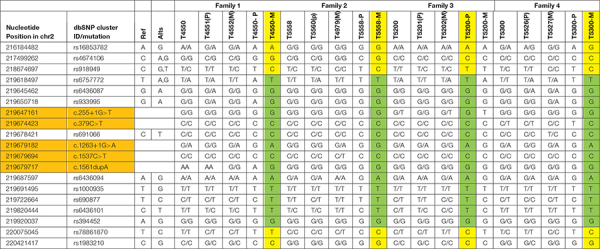

*P, Parternal; M, Maternal; -P, Haplotypes from the father; -M, Haplotypes from the Mother; Marked with yellow and green shading-Haplotypes of CYP27A1 c.1263+1G>A.*

## Results

### Histories and Clinical Assessments of the Patients

**FIGURE 1:**
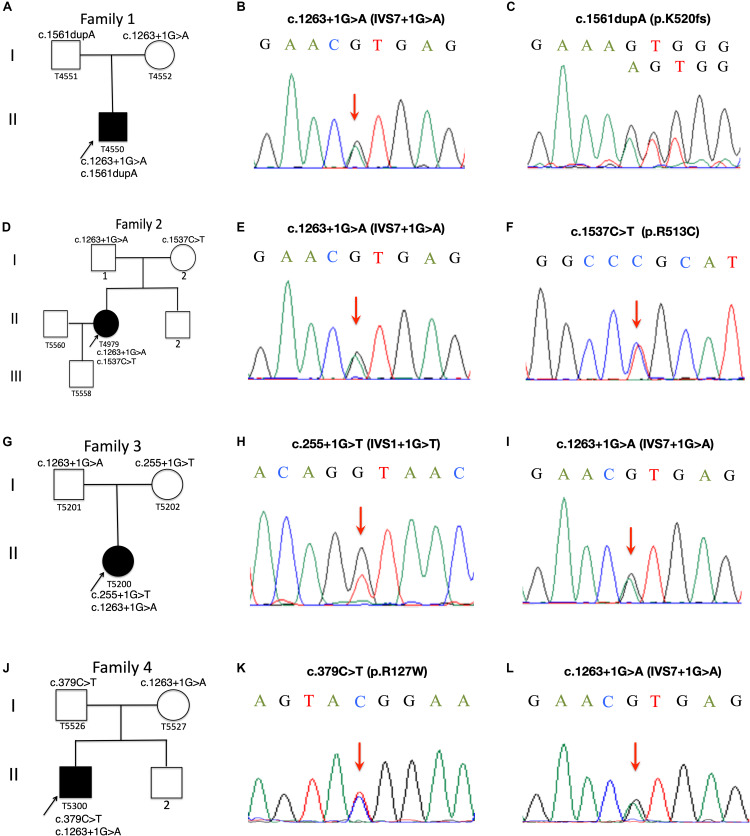
Pedigree charts of Family 1 to Family 4. Squares represent males, circles females; black symbols indicate affected individuals; the arrows indicate the probands, Patient T4550 **(A)**, Patient T4979 **(D)**, Patient T5200 **(G)** and Patient T5300 **(J)**. Sequence chromatograms of the CYP27A1 variants identified in Patient T4550 **(B,C)**, Patient T4979 **(E,F)**, Patient 5200 **(H,I)** and Patient T5300 **(K,L)**.

The pedigrees of the CTX index families were separately shown in Figures 1A,D,G,J. All the 4 probands were born at term following an uneventful pregnancy. There was no known consanguineous marriage with unremarkable histories in these families. The birth history and early developmental milestones of their siblings were normal. The clinical characteristics and biochemical data of these four probands are listed in Table 2.

**TABLE 2:** Clinical findings in 4 CTX patients.

Patient	T4450	T4979	T5200	T5300
Sex	Male	Female	Female	Male
CYP27A1 mutations	c.1263+1G>A	c.1263+1G>A	c.255+1G>T	c.379C>T
	c.1561dupA	c.1537C>T	c.1263+1G>A	c.1263+1G>A
Current age (years)	24	30	34	38
Age at onset (years)	7	8	9	14
Chronic childhood-onset diarrhea	+	+	–	+
Cataracts	–	–	+	+
Tendon xanthomas	–	+	+	–
Foot deformity (pes cavus)	+	–	+	–
**Central Nervous system involvement**				
Ataxia	+	–	+	+
Dysarthria	–	–	+	+
Pyramidal signs/spasticity	+	+	+	+
Seizures	–	–	–	–
Cognitive impairment	+	–	+	–
Mood/affective disorders	–	–	+	+
**Skeletal system involvement**				
Bone fractures	+	–	–	+
MRI Findings				
Bilateral cerebellar lesions	–	–	+	+
Periventricular white hyperintensities	–	–	+	+
Cerebellar atrophy	–	–	–	+
**Laboratory Tests**				
**Plasma cholesterol concentration**				
Total cholesterol (mmol/L)	4.72	5.64	3.92	3.98
High density lipoproteins (mmol/L)	1.01	1.38	2.02	1.15?
Low density lipoproteins (mmol/L)	3.30	3.68	1.77	2.34
Serum triglyceride levels (mmol/L)	0.97	1.47	0.83	1.32

### Patient T4550

**FIGURE 2:**
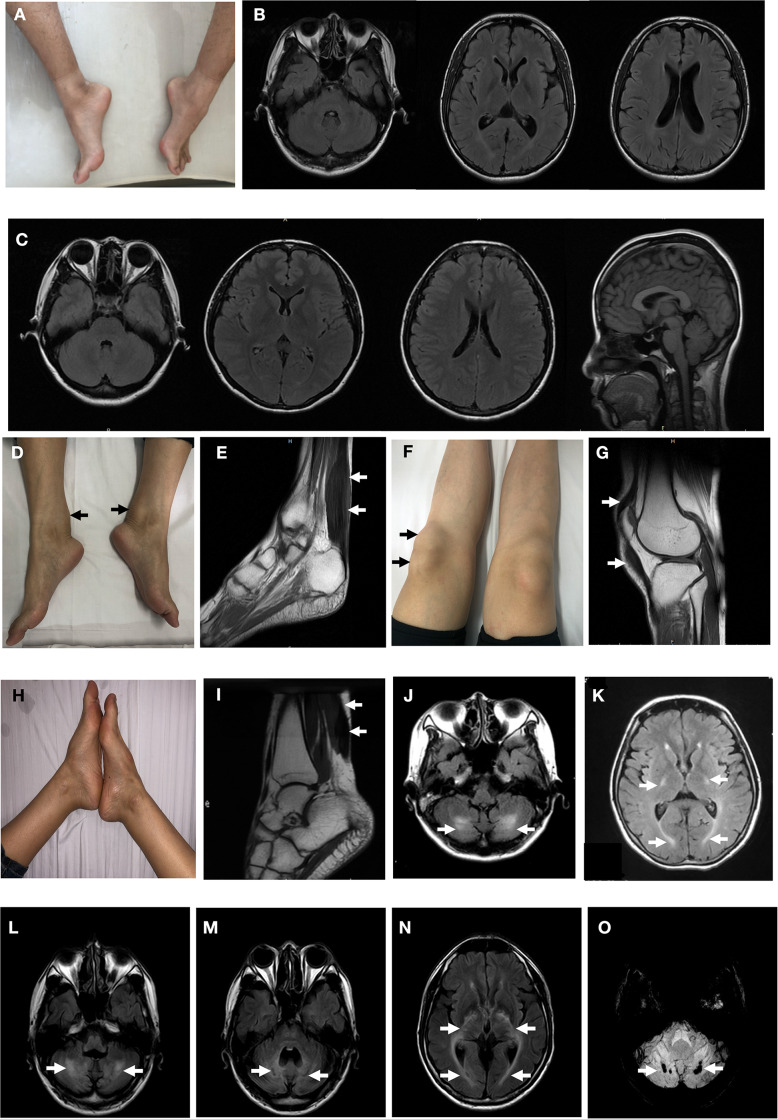
Clinical findings of the probands. Pes cavus deformity of Patient T4550 **(A)**. Brain magnetic resonance MR) of fluid attenuated inversion recovery (FLAIR) images were normal with Patient T4550 and Patient T4979 **(B,C)**. Thickened bilateral Achilles xanthoma and ligamentum patellae of Patient T4979 **(D,F)**. Xanthoma of the Achilles tendon on T2-weighed MRI (arrow) **(E)**. Thickened ligamentum patellae on T2-weighed MRI (arrow) **(G)**. Achilles tendon xanthoma located at right side and bilateral pes cavus of Patient T5200. **(H)** MRI demonstrated fusiform enlargement involving the Achilles tendons of Patient T5200 (arrow) **(I)**. FLAIR MRI of Patient T5200’s brain showed bilateral high-intensity areas in the dentate nuclei **(J)** and periventricular white matter **(K)** (arrow). Patient T5300’s brain MR imaging showed hyperintensity lesions **(L)** and hypointensities **(M)** located at the dentate nuclei (arrow), and periventricular white matter hyperintensities **(N)**. **(O)** Susceptibility-weighted imaging. Multiple microbleeds are seen in the bilateral dentate nuclei of Patient T5300.

Patient T5450 from Family 1 was a 24-year-old man suffering from progressive unsteady gait caused by leg weakness and spasticity. He had a history of chronic infantile diarrhea, with no issues of neonatal jaundice or visual symptoms. He had relatively poor academic performance than his peers since childhood. There were no personality changes noted. The cognitive assessment measured by Mini-mental State Examination (MMSE) was 21. Neurological examination revealed his cranial nerves were normal. The muscle strength of the lower limbs was 4/5. Bilateral deep tendon reflexes were symmetrical and hyperactive. The presence of bilateral positive Babinski reflexes and pes cavus deformity were detected (Figure 2A). The neuroimaging didn’t show any abnormal evidence (Figure 2B). Regarding the laboratory findings, there was no elevation in the levels of serum cholesterol or triglycerid. Exome sequencing revealed a compound heterozygous mutation in the proband, c.1263+1G>A (Figure 1B) in intron 7 and c.1561dupA (p.K520fs) (Figure 1C) in exon 9. Genetic testing of his parents showed a status of asymptomatic carriers of heterozygous mutation.

### Patient T4979

Patient T4979 in Family 2 was a 30-year-old women who initially showed gait disorder with weakness of right lower limb at the age of 28. Two years later, she began to suffered from balance impairment due to progressive spasticity. Tendinous xanthomas located at bilateral ankles and knees progressively enlarged for about 20-year duration (Figures 2D,F). She had no history of chronic diarrhea. Cataract and mental retardation were also absent. On further examination, pyramidal tract signs including bilaterally enhanced deep tenden reflexes and positive Babinski reflexes. The muscle strength of the right lower limb was 4/5. The MRI scan of the brain revealed normal (Figure 2C). T2-weighted MRI scan of the ankles and knees showed low-intensity lesions in the thickened bilateral Achilles tendons and ligamentum patellae (Figures 2E,G). Gene analysis confirmed the detection of a compound mutation including c.1263+1G>A (Figure 1E) in intron 7 and c.1537C>T (p.R513C) (Figure 1F) in exon 9 of CYP27A1 gene. Genetic sequencing revealed a heterozygous carrier for c.1263+1G>A in father and heterozygous carrier state for c.1537C>T in mother.

### Patient T5200

Patient 5200 from Family 3 was a 34-year-old woman who had a poor scholastic performance and strange behavior since childhood, presented with progressive slurred speech and swaying while walking. She was diagnosed with cataracts in both eyes at the age of nine, and then she had cataract surgery. Since last year, she began to unable to walk independently with recurrent falls multiple times due to decreased strength in lower limbs. Clinical examination and MRI scans revealed right Achilles tendon xanthoma and bilateral pes cavus (Figures 2H,I). She had subnormal intelligence, reduced speech fluency, impaired memory and comprehension. The MRI imaging showed symmetrical T2 and FLAIR hyperintense lesions in dentate nuclei (Figure 2J), globus pallidus and periventricular white matter (Figure 2K). The proband carried one novel mutation (c.255+1G>T) (Figure 1H) and one previously reported pathogenic mutation (c.1263+1G>A) (Figure 1I). Genetic screening of her parents revealed both her parents were heterozygous carrier for the variants found in the proband.

### Patient T5300

Patient T5300 in Family 4, a 38-year-old male with a history of chronic childhood-onset diarrhea had a chief complaint of slowly progressive gait disturbance and slurring dysarthria for 4 years. One year ago, he began to rely on a wheelchair for ambulance due to bone fracture. He was diagnosed with bilateral cataracts and surgically corrected at the age of 20. Neurological examinations showed bilateral positive pyramidal signs including hyperactive deep tendon flexes and positive Babinski signs. He swayed slightly when he touched the tip of his nose with index finger. It was difficult for him to cooperate with muscle strength test of his limbs. Laboratory tests revealed the high-density lipoprotein was slightly lower than normal. Brain MRI indicated cerebellar atrophy (Figure 2M), T2 and Flair hyperintense signals in dentate nuclei (Figure 2L), periventricular white matter region and along corticospinal tracts in the posterior internal capsules (Figure 2N). Cerebral microbleeds were seen in susceptibility weighted imaging (SWI) (Figure 2O). The hypointensities lesions in the bilateral cerebellar dentate nuclei were also detected on T1-weighted sequences (Figure 2M). Patient 5300 was identified as a compound heterozygous mutation, c.379C>T (p.R127W) (Figure 1K) in exon 2 and abnormal splicing of c.1263+1G>A (Figure 1L) in intron 7. His parents were confirmed as carriers.

Since c.1263+1G>A were detected in all the probands, 14 SNPs located at upstream and downstream of CYP27A1 c.1263+1G>A mutation were selected to construct haplotype for testing the gene identity by descent, and two generations of four families were tested. Both T4550 and T5300 inherited the mutation with c.1263+1G>A from their mother (T4552 and T5527), while T5200 get the mutation from her father. According to the results of SNP testing, the haplotypes of CYP27A1 c.1263+1G>A were partly identical for the four families (Marked with Green in Table 1).

## Discussion

**FIGURE 3:**
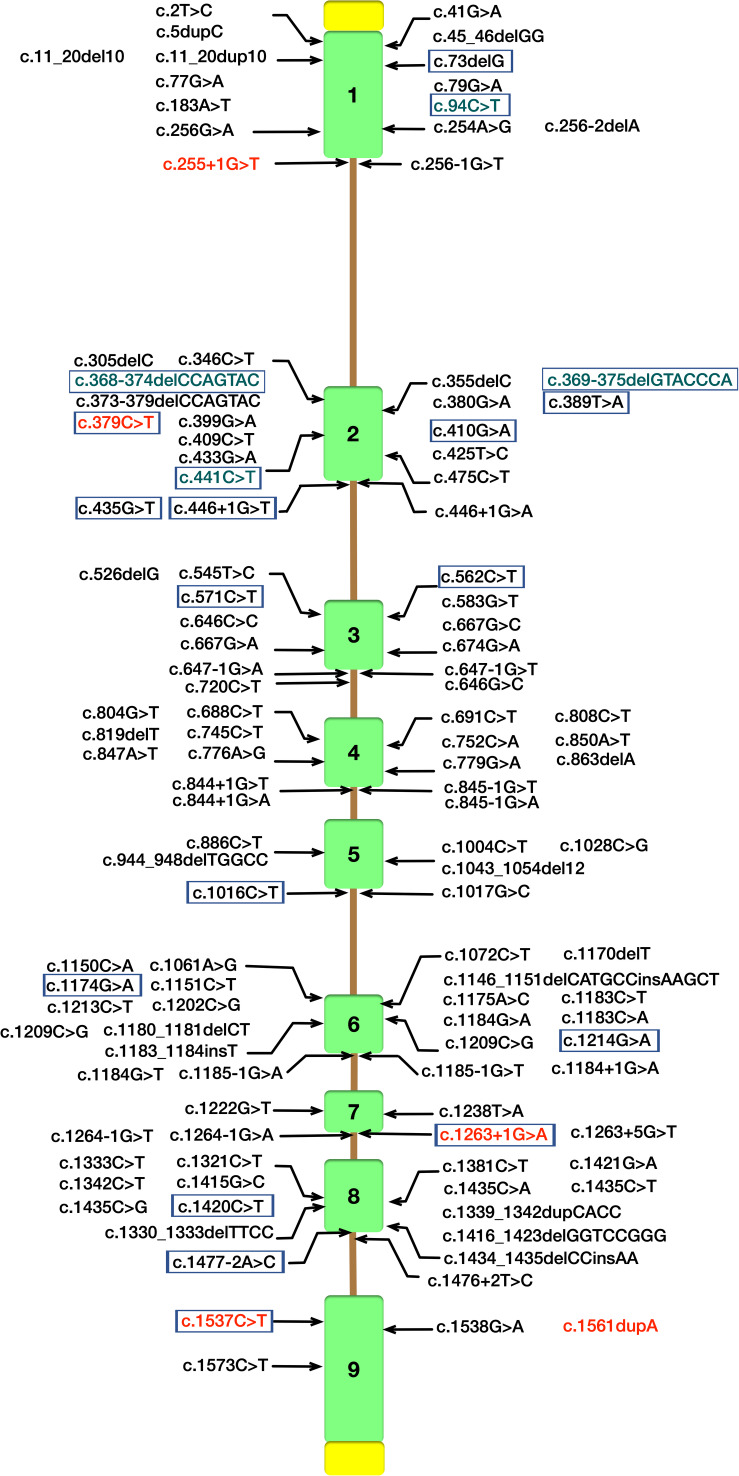
Schematic diagram of *CYP27A1* gene structure with pathogenic mutations according to the Human Gene Mutations Database (HGMD). Mutations identified in our study are in red font. CYP27A1 pathogenic mutations reported in Chinese Han population were marked with purple border (Tao et al., 2019), and the mutations in green font were not included in HGMD.

Cerebrotendinous xanthomatosis is an autosomal recessive lipid storage disease characterized by abnormal deposition of cholestanol and cholesterol in multiple systems (Berginer and Abeliovich, 1981). Since Van Bogaerts published the first case of CTX in 1937 (Gallus et al., 2006) over 118 different mutations implicated in CTX patients have been currently identified in CYP27A1 gene (NM000784.4) according to the Human Gene Mutations Database (HGMD) (Figure 3). CTX is an underdiagnosed and probably treatable disease, thus a detailed appreciation of the phenotypic spectrum and genetic features are crucial for early diagnosis and treatment with chenodeoxycholic acid (CDCA) to ameliorate the life quality of patients.

In our study, all these four probands were compound heterozygote for two CYP27A1 variants, including one mutation in c.1263+1G>A (intron 7) splice site. We also reported two novel likely pathogenic mutations (c.255+1G>T and c.1561dupA) and three pathogenic mutations including c.379C>T, c.1263+1G>A and c.1537C>T previously reported in other case series. The 1263+1 G to A transition in intron 7 leading to exon skipping and a frameshift, together with 1016C?T transition in exon 5 and finally the 5–6 C insertion in exon 1 were found in almost two-thirds of the alleles in the Dutch CTX patients (Verrips et al., 2000a). A nationwide survey recently conducted in Japan reported the most common variant in Japanese CTX patients was c.1214G>A (p.R405Q, 31.6%), followed by c.1421G>A (p.R474Q, 26.3%) and c.435G>T (p.G145 = 15.8%) (Sekijima et al., 2018). In the Chinese population, only 25 patients in 22 families have been described, and the most predominant variants of CYP27A1 gene were c.420G>A and c.379C>T (Tao et al., 2019). Moreover, the mutation with c.1263+1G>A has been previously described in three Chinese families (Guo Hongmei et al., 2017; Li Taoran et al., 2019; Tao et al., 2019). Thus, the 1263+1G>A mutation was commonly seen in Chinese reported case series (7/25, 28%). In this study, a high incidence of CYP27A1 c.1263+1G>A mutation was observed in the genetic testing of sporadic families. The result showed partly identical haplotypes of CYP27A1 c.1263+1G>A in the four families, thus we can not rule out the possibility of the founder effect in very early ancestors. Since the limitation of our study, epidemiologic investigation using high-density SNP array data from a population sample should be conducted to reveal a hotspot phenomenon in Chinese Han population. As far as we know, there are no reports assessing the possible impact of the mutation c.1263+1G>A on gene expression or the splicing process (Lee et al., 2001). Most of the defined mutations were considered as disrupting the heme-binding and adrenodoxin-binding domains which could influence the activity of the enzyme. Some of the mutations which are located the other sites of the protein may recognize a potential substrate-binding or other protein contact site (Bjorkhem and Leitersdorf, 2005).

Cerebrotendinous xanthomatosis is an underdiagnosed disorder lacking of a detailed appreciation of the full phenotypic spectrum. A broad range of neurologic dysfunction have been reported in CTX patients with spastic paraparesis, epilepsy, cerebellar ataxia, dementia and neuropsychiatric disorder (Ly et al., 2014). It was noted that the mutation of CYP27A1 was considered as the pathogenic variants of autosomal recessive hereditary spastic paraplegia (Tao et al., 2019). These four probands predominantly presented with pyramidal signs, nonetheless two of them lacking of the typical symptoms like xanthomas illustrated patients only presented with pure and complicated HSP should not omit the investigation of CYP27A1 gene mutations. CYP27A1 was also identified as a possible candidate gene for amyotrophic lateral sclerosis (Diekstra et al., 2012). CTX may present as progressive upper motor neuron loss such as pure pyramidal signs mimicking a clinical featured symptoms of ALS (Gallus et al., 2006). Hypercholesterolemia and an atherogenic lipoprotein profile having a positive effect on the length of survival in ALS suggested a possible link between ALS and CTX (Chio et al., 2009). Cerebellar signs, especially ataxia, are mostly featured symptoms of CTX patients (Verrips et al., 2000b). The cerebellar involvement caused by abnormal deposition of cholestanol and cholesterol is another reason of ataxia mostly seen in CTX. The reported incidence of “extrapyramidal signs” in CTX is 21–33% (Pilo-de-la-Fuente et al., 2011; Yahalom et al., 2013). Parkinsonism is the mostly reported type of movement disorder followed by dystonia, myoclonus and postural tremor (Stelten et al., 2019). Psychiatric manifestations may emerge during childhood or adolescence as the form of personality disorder, and could be associated with learning difficulties (Fraidakis, 2013). Non-neurological symptoms preceding neurological signs associated with CTX including tendon xanthoma, childhood-onset cataracts are helpful for early diagnosis. Osteoporosis and repeated bone fractures are also commonly seen in CTX patients. Both patient T4550 and patient T5300 had a history of bone fractures because of low bone mass and severe gait disturbances (Stelten et al., 2019). Besides of tendon xanthomas mostly affecting the Achilles tendon, pes cavus deformity detected in two of our patients is another generally consistent feature of CTX (Salen and Steiner, 2017). Several biochemical abnormalities characterize CTX which could aid in differential diagnosis include elevated plasma cholestanol concentration, low to normal plasma cholesterol concentration, increased quantities of bile alcohol and decreased CDCA level (Salen and Steiner, 2017). Though we had no access in evaluating serum profile of cholestanol, no elevation of serum cholesterol or triglyceride has been observed in all the four probands.

MRI imaging studies reveals cerebellar atrophy, white matter signal alterations, and symmetric hyperintensities in the dentate nuclei, globus pallidus, substantia nigra, inferior olives or supratentorial white matter on T2 weighted images (Vanrietvelde et al., 2000). The cerebellar lesions may present as hyperintensities caused by abnormal lipid storage, or appear as hypointensities suggesting vacuolation due to cerebellar degeneration caused by cholestanol-induced apoptosis (Inoue et al., 1999). The spontaneous, round foci of low signal intensity in the cerebellar detected on the susceptibility-weighted imaging (SWI) of patient T5300 were considered as microbleeds (Mignarri et al., 2012). Microbleeds may be caused by advanced age, diabetes mellitus, hypertension, cerebral amyloid angiopathy, traumatic brain injury and primary angiitis of the CNS (Chang et al., 2009). The microbleeds in the dentate nuclei may have relationship with other mechanisms remained unclear leading to neuronal loss.

## Conclusion

In our study, all these four probands were compound heterozygotes for two CYP27A1 variants, including one mutation in c.1263+1G>A (intron 7) splice site, two novel likely pathogenic mutations (c.255+1G>T and c.1561dupA) and three pathogenic mutations including c.379C>T, c.1263+1G>A, and c.1537C>T have been previously reported. The 1263+1G>A mutation was commonly seen in Chinese reported case series (7/25, 28%) and could be a latent hotspot for Chinese CTX mutations. CTX is generally confirmed diagnosis based on clinical findings, neuroimaging, bio-chemical testing and genetic analysis. Early diagnosis of CTX is crucial for early and long-term treatment with CDCA which could be an effective adoption for reversing and preventing related clinical symptoms (Brienza et al., 2015).

## Data Availability Statement

The raw data supporting the conclusions of this article will be made available by the authors, without undue reservation, to any qualified researcher.

## Ethics Statement

The studies involving human participants were reviewed and approved by the Ethics Committee of Ruijin Hospital, Shanghai Jiao Tong University School of Medicine, Shanghai, China. The patients/participants provided their written informed consent to participate in this study. Written informed consent was obtained from the individual(s) for the publication of any potentially identifiable images or data included in this article.

## Author Contributions

JJ: data acquisition, analysis and interpretation of data, and drafting the manuscript. GC: data acquisition and analysis and interpretation of data. JW, XLu, HZ, XLi, ZZ, XS, SW, XQ, JD, and XH: data acquisition. MZ and WX: data acquisition and manuscript revision. LC: funding, study design and conceptualization, data acquisition, analysis and interpretation of data, and manuscript revision. All authors contributed to the article and approved the submitted version.

## Conflict of Interest

The authors declare that the research was conducted in the absence of any commercial or financial relationships that could be construed as a potential conflict of interest.

## Footnotes

1 http://www.ncbi.nlm.nih.gov/snp/
