# Supplementary material for: c.1263+1G>A Is a Latent Hotspot for CYP27A1 Mutations in Chinese Patients With Cerebrotendinous Xanthomatosis
Source: Front Genet. 2020 Jul 1;11:682. doi: 10.3389/fgene.2020.00682 (PMC7342084; doi:10.3389/fgene.2020.00682)
Supplement: Supplementary file 1 [file Table_1.pdf]

Supplementary Table 1. Currently known mutations in *CYP27A1* gene. This list is compiled data from a list of CYP27A1 mutations described according to HGMD(human gene mutation database). All the mutaions are described according to HGVS nomenclature.

No	Nucleotide change	Amino acid change	Variant Class	Reference
1	c.2T>C	p.M1?	DM	Bajaj (2013) J Neurosci Rural Pract **4,** S87
2	c.41G>A	p.R14Q	DM?	Inanloorahatloo (2013) Eur J Med Genet **56,** 655
3	c.77G>A	p.R26K	DM?	Inanloorahatloo (2013) Eur J Med Genet **56,** 655
4	c.79G>A	p.A27T	DM?	Inanloorahatloo (2013) Eur J Med Genet **56,** 655
5	c.183A>T	p.P61=	DM?	Cappi (2016) Transl Psychiatry **6,** e764
6	c.254A>G	p.Q85R	DM	Yanagihashi (2016) BMC Neurol **16,** 21
7	c.256G>A	p.V86M	DM?	Inanloorahatloo (2013) Eur J Med Genet **56,** 655
8	c.346C>T	p.Q116*	DM	Gong (2017) J Pediatr Gastroenterol Nutr **65,** 561
9	c.380G>A	p.R127Q	DM	Gupta (2007) Metabolism **56:** 1248
10	c.379C>T	p.R127W	DM	Kapás (2014) Neuropathol Appl Neurobiol **40:** 345
11	c.389T>A	p.M130K	DM	Gong (2017) J Pediatr Gastroenterol Nutr **65,** 561
12	c.399G>A	p.W133*	DM	Pilo-de-la-Fuente (2011) Eur J Neurol **18,** 1203
13	c.410G>A	p.R137Q	DM	Chen (2017) Metab Brain Dis **32:** 1609
14	c.409C>T	p.R137W	DM	Gupta (2007) Metabolism **56:** 1248
15	c.425T>C	p.L142P	DM	Huijgen (2012) Clin Genet **81,** 24
16	c.433G>A	p.G145R	DM	Gallus (2010) Eur J Neurol **17,** 1259
17	c.475C>T	p.Q159*	DM	Xiong (2015) Science **347:** 1254806
18	c.545T>C	p.I182T	DM?	Traboulsee (2017) Hum Genet **136,** 705

19	c.562C>T	p.R188*	DM	Chen (2017) Metab Brain Dis **32,** 1609
20	c.571C>T	p.Q191*	DM	Huang (2011) Orthopedics **34,** e960
21	c.583G>T	p.E195*	DM	Wakamatsu(1999) J Neurol Neurosurg Psychiatry **67,** 195
22	c.646G>C	p.A216P	DM	Gupta (2007) Metabolism **56:** 1248
23	c.667G>C	p.E223Q	DM	Degos (2016) Orphanet J Rare Dis **11,** 41
24	c.667G>A	p.E223K	DM?	Traboulsee (2017) Hum Genet **136,** 705
25	c.674G>A	p.R225H	DM	Inanloorahatloo (2013) Eur J Med Genet **56,** 655
26	c.688C>T	p.Q230*	DM	Xiong (2015) Science **347:** 1254806
27	c.691C>T	p.R231*	DM	Garuti (1997) J Lipid Res **38,** 2322 Xiong (2015) Science **347:** 1254806
28	c.745C>T	p.Q249*	DM	Verrips (2000) Brain **123,** 908<br>Xiong (2015) Science **347:** 1254806
29	c.752C>A	p.S251*	DM	Gallus (2010) Eur J Neurol **17,** 1259 Xiong (2015) Science **347:** 1254806
30	c.776A>G	p.K259R	DM	Gupta (2007) Metabolism **56:** 1248
31	c.779G>A	p.W260*	DM	Verrips (2000) Brain **123,** 908<br>Xiong (2015) Science **347:** 1254806
32	c.804G>T	p.W268C	DM	Pilo (2011) Muscle Nerve **43,** 531
33	c.808C>T	p.R270*	DM	Sperhake (2000) Int J Legal Med **113,** 110
34	c.847A>T	p.K283*	DM?	Punj (2018) Am J Hum Genet **102,** 1078
35	c.850A>T	p.K284*	DM	Stelten (2018) J Inherit Metab Dis **41:**641
36	c.886C>T	p.Q296*	DM	Gong (2017) J Pediatr Gastroenterol Nutr **65,** 561

37	c.1004C>T	p.A335V	DM	Yoshinaga (2014) Intern Med **53,** 2725
38	c.1016C>T	p.T339M	DM	Schabhüttl (2014) J Neurol **261:** 970
39	c.1028C>G	p.T343R	DM	Pilo-de-la-Fuente (2011) Eur J Neurol **18,** 1203
40	c.1061A>G	p.D354G	DM	Gupta (2007) Metabolism **56:** 1248
41	c.1072C>T	p.Q358*	DM?	Traboulsee (2017) Hum Genet **136,** 705
42	c.1151C>T	p.P384L	DM?	1000 Genomes Project (2010) Nature **467:** 1061
43	c.1150C>A	p.P384T	DM?	Corral(2018) Atherosclerosis **277,** 256
44	c.1175A>C	p.E392A	DM	Alcalay (2009) Mov Disord **24,** 1397
45	c.1174G>A	p.E392K	DM	Zhang (2016) Zhonghua Yi Xue Yi Chuan Xue Za Zhi **33,** 476
46	c.1183C>T	p.R395C	DM	Smalley (2015) Genet Mol Biol **38:** 30
47	c.1184G>A	p.R395H	DM	Gupta (2007)Metabolism **56:** 1248
48	c.1183C>A	p.R395S	DM	Chen (1998) Biochemistry **37**
49	c.1202C>G	p.P401R	DM	Gupta (2007) Metabolism **56:** 1248
50	c.1209C>G	p.N403K	DM	Castelnovo (2003) J Neurol Neurosurg Psychiatry **74,** 1335
51	c.1214G>A	p.R405Q	DM	Abe (2016) J Spinal Cord Med **39:** 726
52	c.1213C>T	p.R405W	DM	Traboulsee (2017) Hum Genet **136:** 705
53	c.1222G>T	p.E408*	DM	von Bahr (2005) J Pediatr Gastroenterol Nutr 40, 481
54	c.1238T>A	p.V413D	DM	Koyama (2012) Clin Neurol Neurosurg **114,** 1021
55	c.1321C>T	p.P441S	DM	Gupta (2007)Metabolism **56:** 1248
56	c.1333C>T	p.Q445*	DM	Xiong (2015) Science **347:** 1254806
57	c.1342C>T	p.R448C	DM	Alazami (2015) Cell Rep

				**10,** 148
58	c.1381C>T	p.Q461*	DM	Lee (2001) J Lipid Res **42,** 159 Schneider (2010) Orphanet J Rare Dis **5:** 27
59	c.1415G>C	p.G472A	DM	Verrips (2000) Brain **123,** 908
60	c.1421G>A	p.R474Q	DM	Stelten (2018) J Inherit Metab Dis **41:** 641
61	c.1420C>T	p.R474W	DM	Kim (1994) J Lipid Res **35,** 1031 Gupta (2007) Metabolism **56:** 1248
62	c.1435C>T	p.R479C	DM	Mandrile (2014) Neurol Sci **35:** 1303
63	c.1435C>G	p.R479G	DM	Guyant-Marechal (2005) Am J Med Genet A **139A,** 114
64	c.1435C>A	p.R479S	DM	Jiao (2018) J Intern Med **283,** 604
65	c.1537C>T	p.R513C	DM	Chen (2017) Metab Brain Dis **32,** 1609
66	c.1538G>A	p.R513H	DM	Ginanneschi (2013) J Neurol **260,** 268
67	c.1573C>T	p.Q525*	DM	Pilo (2011) Muscle Nerve **43,** 531
68	c.256-1G>T	IVS1 as G-T -1	DM	Smalley (2015) Genet Mol Biol **38,** 30
69	c.435G>T	IVS2 ds G-T +12	DM	Chen (1998) Biochemistry **37,** 4420
70	c.446+1 G>A	IVS2 ds G-A+1	DM	Verrips (2000) Brain **123,** 908
71	c.446+1 G>T	IVS2 ds G-T+1	DM	Alhariri (2017) Am J Med Genet A **173,** 2275
72	c.647-1 G>A	IVS3 as G-A-1	DM?	Appadurai (2015) Mol Genet Metab **116,** 298
73	c.647-1 G>T	IVS3 as C-T -1	DM	Gallus (2010) Eur J Neurol **17,** 1259 Xiong (2015) Science **347:** 1254806
74	c.720C>T	IVS3 as C-T +74	DM?	Corral (2018) Atherosclerosis **277,** 256
75	c.646G>C	IVS3 ds G-C-1	DM	Garuti (1996) J Lipid Res **37,** 1459 Xiong (2015) Science **347:** 1254806

76	c.845-1G>A	IVS4 as G-A-1	DM	Leitersdorf (1993) J Clin Invest **91,** 2488<br>Xiong (2015) Science **347:** 1254806
77	c.845-1G>T	IVS4 as G-T-1	DM	Lee (2001) J Lipid Res **42,** 159<br>Xiong (2015) Science **347:** 1254806
78	c.844+1G>A	IVS4 ds G-A+1	DM	Verrips (1997) Hum Genet **100,** 284<br>Xiong (2015) Science **347:** 1254806
79	c.844+1G>T	IVS4 ds G-T+1	DM	Pilo-de-la-Fuente (2011) Eur J Neurol **18,**1203
80	c.1016C>T	IVS5 ds C-T-2	DM	Lee (2001) J Lipid Res **42,** 159
81	c.1017G>C	IVS5 ds G-C-1	DM	Wallon (2010) Clin Neuropathol **29,** 361
82	c.1185-1G>A	IVS6 as G-A-1	DM	Smalley (2015) Genet Mol Biol **38,** 30
83	c.1185-1G>T	IVS6 as G-T-1	DM	Mak (2004) Mol Genet Metab **81,** 144
84	c.1184G>T	IVS6 ds G-T-1	DM	Lee (2001) J Lipid Res **42,** 159
85	c.1184+1G>A	IVS6 ds G-A+1	DM	Garuti (1997) J Lipid Res **38,** 2322<br>Xiong (2015) Science **347:** 1254806
86	c.1264-1G>A	IVS7 as G-A-1	DM	Garuti (1997) J Lipid Res **38,** 2322<br>Xiong (2015) Science **347:** 1254806
87	c.1264-1G>T	IVS7 as G-T-1	DM	Lee (2001) J Lipid Res **42,** 159
88	c.1263+1G>A	IVS7 ds G-A+1	DM	Xiong (2015) Science **347:** 1254806
89	c.1263+5G>T	IVS7 ds G-T+5	DM	Garuti (1997) J Lipid Res **38,** 2322
90	c.1477-2A>C	IVS8 as A-C-2	DM	Chen (2017) Metab Brain Dis **32,** 1609
91	c.1476+2T>C	IVS8 ds T-C+2	DM	Degos (2016) Orphanet J Rare Dis **11,** 41
92	c.11_20del10	p.(Leu4Argfs*3)	DM	Lee (2001) J Lipid Res **42,** 159

93	c.45_46delGG	p.(Ala16Argfs*164)	DM	Koge (2016) Rinsho Shinkeigaku **56,** 667
94	c.73delG	p.(Ala25Profs*33)	DM	Tian (2011) BMC Neurol **11,**130
95	c.256-2delA	Not yet available	DM	Degos (2016) Orphanet J Rare Dis **11,** 41
96	c.305delC	p.(Pro102Leufs*5)	DM	Wang (2006) Proteomics **6,** 1029
97	c.355delC	p.(Arg119Glyfs*24)	DM	Leitersdorf (1994) Am J Hum Genet **55,** 907
98	c.373_379delCCAGTAC	p.(Pro125Glyfs*16)	DM	Tian (2011) BMC Neurol **11,** 130
99	c.526delG	p.(Asp176Metfs*6)	DM	Dutta (2015) Mol Genet Metab Rep **3:** 33
100	c.819delT	p.(Asp273Glnfs*13)	DM	Leitersdorf (1993) J Clin Invest **91,** 2488 Yahalom (2013) ClinvNeuropharmacolv**36:** 78
101	c.863delA	p.(Glu288Glyfs*57)	DM	Gallus (2010) Eur J Neurol **17,** 1259
102	c.944_948delTGGCC	p.(Leu315Glnfs*15)	DM	Lamon-Fava (2002) Clin Genet 61, 185
103	c.1043_1054del12	p.(Leu348_Ser352delinsPro)	DM	Pilo (2011) Muscle Nerve **43,** 531
104	c.1170delT	p.(Lys391Afgfs*17)	DM	Abdel-Hamid (2017) Metab Brain Dis **32,** 311
105	c.1180_1181delCT	p.(Leu394Alafs*18)	DM	Lee (2001) J Lipid Res **42,** 159
106	c.1330_1333delTTCC	p.(Phe444Serfs*6)	DM	Schneider (2010) Orphanet J Rare Dis **5,** 27
107	c.1416_1423delGGTCCGGG	p.(Val473Leufs*45)	DM	Pilo (2011) Muscle Nerve **43,** 531
108	c.5dupC	p.(Ala3Cysfs*178)	DM	Segev (1995) Hum Genet **95,** 238
109	c.11_20dup10	p.(Arg8Glyfs*176)	DM	Teszas (2006) Arch Ophthalmol **124,** 1490
110	c.1183_1184insT	p.(Arg395Leufs*18)	DM	Giraldo-Chica (2015) Biomedica **35,** 563
111	c.1339_1342dupCACC	p.(Arg448Profs*74)	DM	Sasamura (2018) Intern Med **epub,** epub
112	c.1146_1151delCATGCCins AAGCT	p.(His382Glnfs*26)	DM	Suh (2012) Eur J Med Genet **55,** 71
113	c.1434_1435delCCinsAA	p.(Arg479Ser)	DM	Hansson (2007) J Intern

				Med **261,** 504
114	2kb IVS6-3’ UTR	Not yet available	DM	Garuti (1996) J Lipid Res **37,** 662
115	Ins C nt.26,C-T nt.1172,P351L	/	DM	Verrips (2000) Brain **123,** 908
116	c.368-374delCCAGTAC	/	DM/	Tao(2019) Orphanet Journal of Rare Diseases,14,282
117	c.369-375delGTACCCA	/	DM?	Tao(2019) Orphanet Journal of Rare Diseases,14,282
118	c.441C>T	p.E147D	DM?	Tao(2019) Orphanet Journal of Rare Diseases,14,282
